# Survey of UK horse owners’ knowledge of equine arboviruses and disease vectors

**DOI:** 10.1136/vr.104521

**Published:** 2018-05-15

**Authors:** Gail Elaine Chapman, Matthew Baylis, Debra C Archer

**Affiliations:** 1 Epidemiology and Population Health, Institute of Infection and Global Health, University of Liverpool, Liverpool, UK; 2 Health Protection Research Unit in Emerging and Zoonotic Infections, University of Liverpool, Liverpool, UK

**Keywords:** arthropod-borne infections (arboviruses), disease surveillance, entomology, west nile fever virus, African horse sickness

## Abstract

Increased globalisation and climate change have led to concern about the increasing risk of arthropod-borne virus (arbovirus) outbreaks globally. An outbreak of equine arboviral disease in northern Europe could impact significantly on equine welfare, and result in economic losses. Early identification of arboviral disease by horse owners may help limit disease spread. In order to determine what horse owners understand about arboviral diseases of horses and their vectors, the authors undertook an open, cross-sectional online survey of UK horse owners. The questionnaire was distributed using social media and a press release and was active between May and July 2016. There were 466 respondents, of whom 327 completed the survey in full. High proportions of respondents correctly identified photographic images of biting midges (71.2 per cent) and mosquitoes (65.4 per cent), yet few were aware that they transmit equine infectious diseases (31.4 per cent and 35.9 per cent, respectively). Of the total number of respondents, only 7.4 per cent and 16.2 per cent correctly named a disease transmitted by biting midges and mosquitoes, respectively. Only 13.1 per cent and 12.5 per cent of participants identified specific clinical signs of African horse sickness (AHS) and West Nile virus (WNV), respectively. This study demonstrates that in the event of heightened disease risk educational campaigns directed towards horse owners need to be implemented, focussing on disease awareness, clinical signs and effective disease prevention strategies.

## Introduction

Globalisation and climate change have led to increasing concern over the risk of arthropod-borne virus (arbovirus) outbreaks in northern Europe.^1 2^ There is evidence of increasing risk to equids in the UK and other areas of Europe that are currently free from equine arboviruses, and surveys of equine premises in the UK have demonstrated the presence of several species of mosquitoes that are known to be vectors of equine arboviruses.^3^ Biting midges, which act as vectors for African horse sickness (AHS) are known to be widespread on UK equine premises.^4^

Arboviral disease can appear and spread very rapidly, and new viruses may appear, as demonstrated by the Schmallenberg outbreak in ruminants that occurred in 2011 and spread across the UK.^5^ It has been stated that horse owner awareness of clinical signs would be key in limiting an outbreak of AHS in the UK.^6^ Early recognition of an equine arboviral disease and measures to limit its spread are essential in minimising rates of mortality and morbidity, and duration of a disease outbreak. For example, an outbreak of AHS that first started in central Spain in 1987 was initially not recognised, as local veterinary surgeons and horse owners were not aware of the key clinical signs of disease. The resultant disease outbreak spread over three countries and lasted over four years resulting in the death of approximately 1400 equids in Spain alone.^7^ An outbreak of AHS in Asia caused the deaths of over 300,000 horses from 1959 to 1961.^8^ It has been estimated that the cost to the UK government of an AHS outbreak could be £4–35 million, depending on the scale the outbreak,^9^ and in the Netherlands, total costs have been estimated at €272–516 million.^10^ In addition to AHS, horses in northern Europe are at potential risk from West Nile virus (WNV)^11^ and possibly other mosquito-borne arboviruses, such as Eastern equine encephalitis virus (EEEV), Western equine encephalitis virus (WEEV), Venezuelan equine encephalitis virus (VEEV) and Japanese encephalitis virus (JEV).^1 12^

Assessment of horse owners’ knowledge about clinical signs of arboviral diseases, how disease is spread and whether vaccines are available to control or limit disease spread are important factors to consider as owner compliance with preventive and control measures would be required in the event of a disease outbreak.^13–15^ This information could inform education strategies directed at horse owners about the risk of disease and how to recognise clinical signs in affected horses, and could assist early recognition of disease, particularly in situations of heightened disease risk.

The aim of this study was to investigate UK horse owners’ knowledge about insects that bite horses, methods by which insects are prevented from biting horses and knowledge of insect-borne viral diseases that affect horses. A priori, the authors hypothesised that horse owners would have limited knowledge of insect-borne diseases of horses and would use a variety of bite-protection methods with spray-repellents being most popular.

## Methods

### Data collection

A cross-sectional survey (Supplementary item 1) of UK horse owners/carers was conducted using an online questionnaire tool (Survey Monkey, Survey Monkey, Palo Alto, California, USA). To be included in the study, participants had to be currently caring for one or more horses in the UK and be over 18 years in age. The questionnaire was available online from 13 May – to 27 July 2016. The survey was posted on the study website and was promoted via social media using Facebook and Twitter and in a university press release. The survey link was posted to general equine discussion forums and promoted through relevant organisations such as the British Horse Society and local groups of The Pony Club, through equine charities such as The Horse Trust and The Donkey Sanctuary and through social media, such as Facebook, Twitter and websites, as deemed appropriate by the administrators of these media. Veterinary practices also posted promotion of the survey. Further dissemination relied on users sharing posts about the survey.

10.1136/vr.104521.supp1 Supplementary file 1 Item Questionnaire

The survey focused on mosquito and midge-borne diseases of horses and covered the following themes: awareness and knowledge of arthropod-borne diseases of horses worldwide; attitude to and knowledge of insects on the premises where their horse is kept; opinions about vaccination and bite protection methods, including use of insect repellents. The questionnaire was piloted with 10 horse owners and amended based on their responses and comments before release.

## Data analyses

A descriptive analysis of the responses to each question was performed. Respondents were not forced to respond to any single question so in calculating percentages the denominator used (unless stated otherwise) was the number of participants who answered subsequent questions. Therefore, in the case of participants who left an answer blank but answered further questions it was inferred that the question had been left intentionally blank. For open-ended questions inductive coding (application of categories produced by looking for patterns in the responses, rather than categories decided on before release of the survey) was applied before analysis.^16^ Coding was undertaken solely by the first author. Comparisons between two sample proportions were tested using a two-sample z-test.^17^

## Results

In total, 466 surveys were completed and 70.2 per cent (327) had been completed in full (defined as at least one question completed on all pages of the survey, and completion of all questions which could not be answered "I do not know"). All responses were analysed.

### Ability to identify insects, and insect nuisance

A total of 365 respondents (98.6 per cent, n=370) stated they were aware of biting insects on the premises where their horse was kept (Table 1), including five respondents who additionally named arthropods that are not biting flies, such as spiders and hornets. In the free text answers, six respondents named ticks. Overall, 95.1 per cent (352/370) of respondents named at least one biting fly (mosquito, midge, stable fly, horse fly, gnat). The majority of respondents (331/465, 71.2 per cent) were able to correctly identify a photographic image as a midge, and only 11 (2.4 per cent) reported not having seen this insect before. Over half of respondents (284/434, 65.4 per cent) were able to correctly identify a photographic image as a mosquito and only 14 (3.2 per cent) reported not having seen one before. Of those respondents who answered ‘yes’ to whether they were aware of mosquitoes on their yard, 79.5 per cent (105/132) were able to correctly identify mosquitoes, compared with 57.9 per cent (135/233) of respondents who were not aware of mosquitoes on their yard (P<0.001). A stable fly was correctly identified by 56.9 per cent (235/413) of respondents and 49.4 per cent respondents (195/395) correctly identified a horse fly.

**TABLE 1:** Number of respondents who are aware of biting insects present on the premises where they keep their horse(s), and the proportion of those who are aware of the presence of these insects correctly identifying images of the insects

Insect	Respondents reporting they are aware of this insect on the yard where they keep their horse(s) (n=367)	Number of these respondents correctly identifying insect
Mosquito	132 (36.0%)	105 (79.5%) 114* (86.4%)
Biting midge	322 (87.7%)	239 (74.2 %)
Stable fly	132 (36.0%)	100 (75.8 %)
Horse fly	294 (80.1%)	155 (52.7 %)

For each image options given were mosquito, biting midge, stable fly, horse fly, gnat, "I have never seen this before" and "I do not know".

*Including those using the term ’gnat' to describe a mosquito in the image.

**FIG 1:**
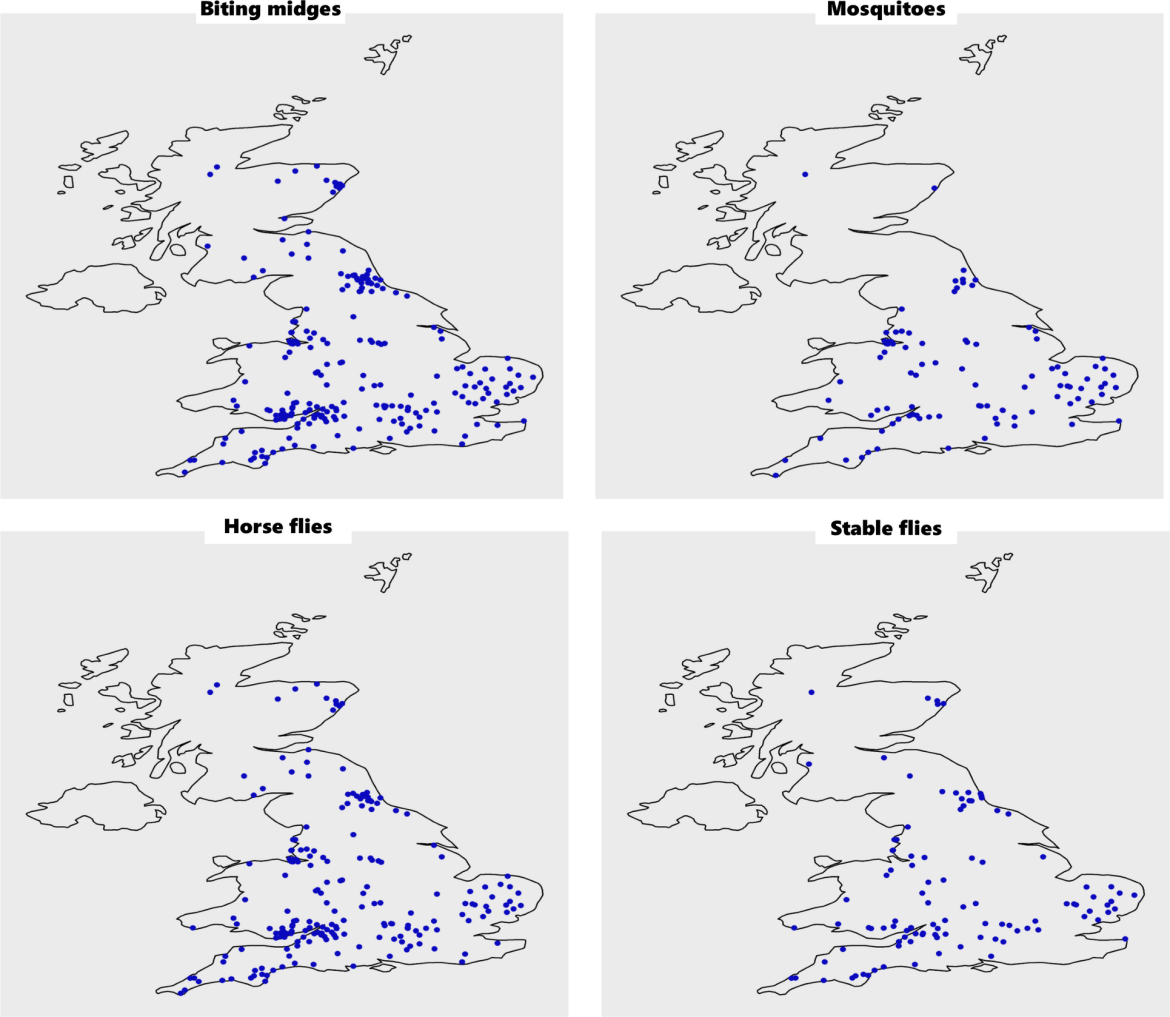
Geographical location of participants who reported that they were aware of each type of fly (and provided postcode or town).

Overall, 27.0 per cent (99/367) respondents stated they were unaware of mosquitoes and 6.8 per cent (25/367) of biting midges, on their yard. Fig 1 shows the distribution of participants who were aware of different biting flies on their horse’s yard and provided postcode data. Participants were asked if they felt the insects caused a problem, not if they specifically caused a problem to the horse. Of 132 respondents who said they were aware of mosquitoes on their yard, 15 (11.4 per cent) stated they did not cause a problem, 70 (53.0 per cent) stated they caused a minor problem and 47 (35.6 per cent) considered that they caused a moderate or major problem. Of respondents who were aware of biting midges on their yard, only 2.5 per cent of participants (8/322) stated they caused no problem, while 65.5 per cent of (211/322) participants felt they caused a moderate or major problem.

### Knowledge about insects and equine disease

**FIG 2:**
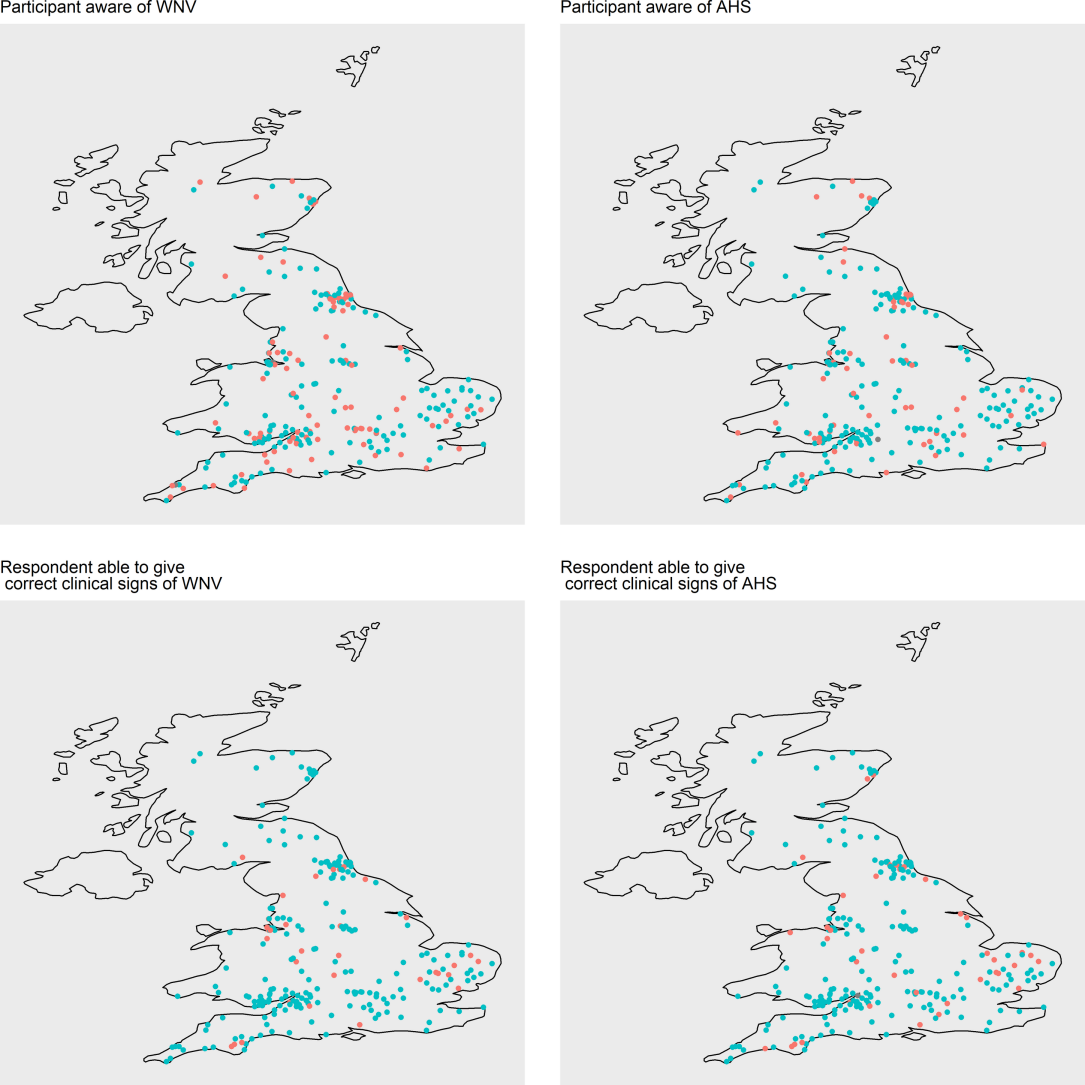
Geographical location of participants who answered questions about their knowledge of West Nile virus (WNV) and African horse sickness (AHS) (and provided postcode or town). Red points correspond to ‘yes’ and blue points correspond to ‘no’.

Of 331 respondents who correctly identified biting midges, 31.4 per cent (104/331) stated that this type of insect could transmit infectious diseases to horses. Of respondents who correctly identified mosquitoes, 40.5 per cent (115/284) believed they only caused allergic disease and 35.9 per cent (102/284) believed that they transmitted infectious diseases in horses worldwide. However, only 7.4 per cent (n=27) of all respondents (366) were able to correctly name a midge-borne disease and 16.2 per cent (59/365) a mosquito-borne disease of horses. When asked to state any infectious disease transmitted by biting midges, to horses worldwide, only 27 of 366 respondents (7.4 per cent) named African horse sickness. Other responses to this question were sweet itch (6.6 per cent; n=24), bluetongue (n=3), malaria (n=1), sarcoids (n=5) and WNV (n=2). When asked to state a mosquito-borne disease of horses, 59 out of 365 respondents (16.2 per cent) correctly identified at least one disease. Most of the correct answers stated WNV only and a small number of respondents (2.7 per cent, 10/365) cited other encephalitides including ‘equine encephalitis’ (n=5), EEEV (n=4) and WEEV (n=4). None of the respondents mentioned VEEV, Murray valley encephalitis (MVE), Ross River fever or other mosquito-borne viruses. AHS was believed to be transmitted by mosquitoes by 9.3 per cent (34/365) of respondents and 9.9 per cent (36/365) cited that mosquito-borne diseases of other species affected horses. These were predominantly human diseases including malaria, Zika, Q-fever and dengue. ‘Malaria’ was cited as a disease of horses transmitted by mosquitoes by 7.9 per cent (29/365) of participants. However, it should be noted that equine piroplasmosis, a tick-borne disease, is also known as equine malaria.^18^ Significantly more respondents were aware of AHS (72.5 per cent, 261/360) compared with WNV (60.5 per cent, 219/362) (P=0.002). Fig 2 shows that the geographical locations of those who were aware of WNV and AHS were not distributed in one particular region. When asked specifically if WNV can affect horses, 83.1 per cent (182/219) of those who responded to this question stated ‘yes’.

### Consequences of an equine arbovirus outbreak and clinical signs of disease

A summary of responses to statements about the possible consequences of an outbreak of either AHS or WNV in the UK is provided in Table 2. When asked to list clinical signs of WNV only, 21.1% (n=69) of all participants responded, despite 327 respondents submitting this page of the survey. Of those who responded, 59.4% (n=41) listed correct clinical signs such as neurological abnormalities, and 17.3% (n=12) stated non-specific signs, which were coded as pyrexia, depression and inappetence, and two respondents mentioned only ‘flu-like symptoms’. Of all survey participants completing this page, the proportion who stated neurological signs in relation to WNV was 12.5 per cent (41/327).

**TABLE 2:** Number of participant responses (%) to statements regarding the consequences of an outbreak of West Nile virus (WNV) or African horse sickness (AHS) in the UK

Statement	WNV			AHS		
	True	False	I do not know	True	False	I do not know
Disease could spread rapidly throughout the UK (respondents: WNV 326; AHS 326)	157 (48.1%)	16 (4.9%)	153 (46.9%)	183 (56.1%)	15 (4.6%)	128 (39.3%)
Many horses could become ill (327; 326)	181 (55.4%)	14 (4.3%)	132 (40.4%)	221 (67.8%)	6 (1.8%)	99 (30.4%)
Horses could die from the disease (328; 328)	173 (52.7%)	7 (2.1%)	148 (45.1%)	218 (66.5%)	0 (0%)	110 (33.5%)
Lots of horses * (more than 1000) could die from the disease (327; 324)	101 (30.9%)	24 (7.3%)	202 (61.8%)	139 (42.9%)	10 (3.1%)	175 (54.0%)
The government would ban movement of horses in affected areas (327; 329)	120 (36.7%)	22 (6.7%)	185 (56.6%)	157 (47.7%)	25 (7.6%)	147 (44.7%)
A vaccination campaign would be necessary to prevent further spread (327; 329)	99 (30.3%)	19 (5.8%)	209 (63.9%)	131 (39.8%)	24 (7.3%)	174 (52.9%)
Vaccination could be done immediately to protect horses (325; 328)	56 (17.2%)	37 (11.4%)	232 (71.4%)	52 (15.9%)	53 (16.2%)	223 (68.0%)

*Participants were provided with the information that there are approximately 900,000 horses in the UK.

When asked to list the clinical signs of AHS, only 22.9 per cent (n=75) of survey participants responded, out of 327 submitting this page of the survey. Of these, three responded that they were not aware of the clinical signs and 43 (13.1 per cent of 327) provided at least one correct and specific clinical sign of AHS, such as foaming at the nostrils, respiratory compromise or facial swelling. Again, the geographical distribution of those demonstrating knowledge of clinical signs for either disease was unremarkable (Fig 2). Only 10 respondents (3.1 per cent) stated death or collapse, despite the high mortality caused by this disease.

### Vaccination

Participants were asked if their horses were currently vaccinated against influenza, tetanus or other diseases. Overall, 97.9 per cent (320/327) responded their horses were vaccinated for tetanus and 88.1 per cent (288/327) for influenza. Only 16 stated their horse was vaccinated against other diseases, including equine herpes virus and grass sickness. One respondent stated their horse was vaccinated against WNV. In the event of a disease outbreak of WNV, most respondents to this question answered that they would have their horse(s) vaccinated against it (80.1 per cent, 262/327), 57 respondents (17.4 per cent) were unsure and 8 respondents (2.4 per cent) would not vaccinate. Participants were asked to write brief comments about factors that would make them less likely to choose vaccination for WNV. Themes included concerns about side effects and efficacy (51.4 per cent; 76/148 respondents), lack of risk of disease (27.7 per cent; 41/148) and cost (14.9 per cent; 22/148). A few respondents (9.5 per cent; 14/148) stated that there was nothing that would prevent them vaccinating their horse(s) in this situation. Of the five respondents who said they would not vaccinate their horses, four were concerned about side effects. Of the 43 respondents who were unsure about vaccinating and gave reasons, 55.8 per cent (n=24) cited potential lack of risk as a barrier, while 41.9 per cent (n=18) cited the balance between efficacy and side effects. Respondents who stated that they vaccinate their horse(s) against influenza were more likely to have them vaccinated against WNV, compared with those who do not vaccinate against influenza (P<0.001). Due to the lack of a commercially available vaccine, the authors did not ask about vaccination against AHS in the current study.

### Use of bite protection methods

**FIG 3:**
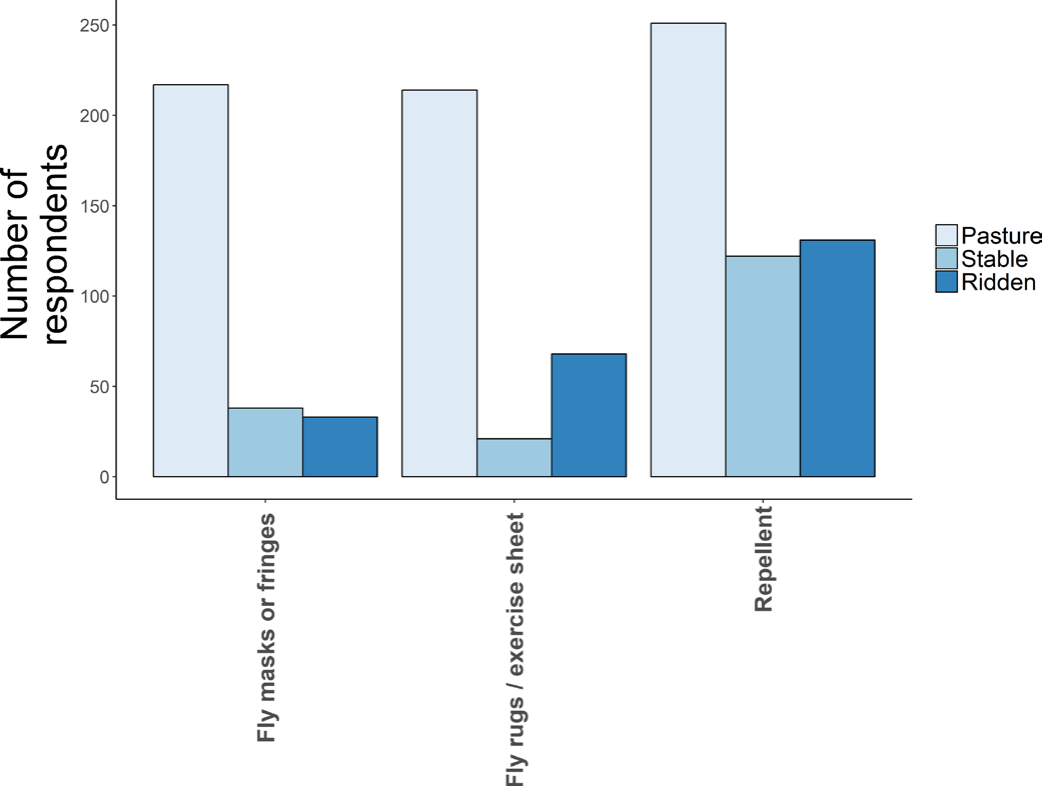
Number of respondents using insect bite protection methods, in the stable, at pasture and when riding their horse.

Participants were asked if they used repellents, fly masks or rugs at pasture, in the stable or when ridden (Fig 3). Most respondents (90.2 per cent; 284/315) stated that they used repellents, 69.5 per cent (219/315) used fly rugs and 71.7 per cent (226/315) used fly masks or fringes. In addition, 10.5 per cent (33/315) of respondents used stable barriers such as fly mesh to prevent insects entering and 35.2 per cent  (111/315) used wash-in insecticides on their horse, such as ‘Deosect’ (cypermethrin 0.1 per cent w/v).

Participants were asked to state which insect repellents they used on their horse(s). They were also asked about alternative bite protection methods in a subsequent question in which some respondents cited products which are believed to have a repellent effect. Respondents’ answers were cross-checked between the two questions to prevent double counting. There were 252 respondents in total: N,N-diethyl-meta-toluamide (DEET) and citronella were the most popular insect repellents used (Table 3). Other methods respondents used to try to reduce insect bites included feeding garlic (n=15), turmeric (n=1) or yeast (n=1), turning horses out at specific times of the day (n=2), use of insecticide or repellent impregnated tags (n=5), fly traps in stables and fields (n=4), permethrin impregnated rugs (n=1) and fans in stables (n=1). When asked an open question about comparative effectiveness of bite-protection methods against all of the biting flies listed, 15 of 46 (32.6 per cent) respondents stated specifically that nothing seemed to work against horse flies.

**TABLE 3:** Insect repellents used on horses as reported by study respondents, and evidence for repellent efficacy in studies on humans

Ingredient/repellent type	Total responses (% of participant responses*) (n=252)	Evidence of repellency against mosquitoes	Evidence of repellency against biting midges
N,N-Diethyl-meta-toluamide (DEET) (including Power Phaser)	89 (35.3%)	Yes^28 35^	Yes^36 37^
Citronella	48 (19.0%)	Variable^35^	No^31^
Power Phaser (DEET and IR3535)	36 (14.3%)	Yes^35^	Yes^37^
NAF product (active ingredient not identified)	25 (9.9%)	–	–
Home made	25 (9.9%)	–	–
Citridiol (also known as oil of lemon eucalyptus, citriodora, PMD)	24 (9.5%)	Yes^35 38^	Yes^39^
Neem oil	19 (7.5%)	Yes^38^	Yes^40^
Avon skin so soft (citronellol)	19 (7.5%)	No	No
Icaridin	8 (3.2%)	Yes†^29 35^	Yes^41^
Tri-tec (cypermethrin and pyrethrins)‡	3 (1.2%)	–	–
Coopers fly repellent (permethrin and citronellol)‡	5 (2.0%)	–	–
Unintelligible	25 (9.9%)	–	–

*Participants who did not respond to this question, or did not state that they used repellents were not included.

†Studies on both horses and humans.

‡Usefulness of topical insecticides is unclear because of their inability to prevent blood-feeding, although treatment of infected horses may subsequently kill vectors that have blood-fed.^42^

PMD, p-Menthane-3,8-diol.

### Sources of information

When participants were asked where they would seek information about insect control, 65.1  per cent (203/312) stated that they would seek advice from their veterinary surgeon and 55.8 per cent (174/312) stated they would use internet sources. Other common answers included tack shop staff (28.2 per cent; 88/312) and other people keeping horses at the same premises (29.5 per cent; 92/312). When asked if a disease outbreak might motivate them to seek information from a different source, 295 participants responded and 63.0 per cent (186/295) stated ‘yes’ to this question. A total of 85.3 per cent (266/312) said that they would seek information about insect control from a veterinary surgeon in the event of an outbreak of disease in the UK. Of the respondents stating they would change or add to sources of information, 17.2 per cent (32/186) expected to be able to obtain information from governmental or industry sources. A small number of respondents (n=6) mentioned they expected the government to issue specific guidelines on insect control in the event of an outbreak.

## Discussion

This survey provides important evidence that UK horse owners currently have poor awareness of equine arboviral diseases, including clinical signs of disease, consequences and controls that might be imposed in the event of a disease outbreak. Given the increasing risk of an equine arboviral disease occurring in northern Europe and potentially the UK, it is important that the veterinary profession has a good understanding of horse owners’ level of knowledge about clinical signs of disease and ways in which specific equine arboviral diseases are spread and controlled. The profession should be able to provide correct, current, evidence-based information to horse owners in the event of an outbreak of arboviral disease.

*Culicoides* biting midges can induce insect-bite hypersensitivity (‘sweet-itch’) in horses,^19^ a common disease in the UK.^20^ Therefore, horse owners might be expected to have knowledge about how to identify and control biting midges. Most study respondents were able to correctly identify a range of flies that bite horses, including midges and mosquitoes, but few horse owners (around a third) were aware that biting midges and mosquitoes could transmit diseases to horses.

Horse owners’ knowledge regarding equine disease transmitted by mosquitoes and midges was poor: many respondents were aware of AHS, WNV, WEE or EEE or ‘equine encephalitis’ but none mentioned VEEV, MVE, Ross River fever or other less well-known arboviruses. Many respondents were also unaware of likely consequences of an outbreak of WNV or AHS. For control of AHS in accordance with the Disease Control Strategy of Great Britain, vaccination during an outbreak and banning horse movements are preferable to culling (except for clinically affected animals). Only 42.9 per cent of respondents answered ‘true’ for the statement that ‘lots of horses could die from AHS’ and just under 70% per cent believed that ‘many horses could become ill’ (Table 2). Just under half of respondents were aware that movement bans may be implemented during an AHS outbreak. These results indicate a lack of awareness of the potentially devastating consequences of an AHS outbreak, to both equine welfare and the equine industry as a whole. Over a third of respondents in this study believed that the government would ban horse movements in the event of an outbreak of WNV, and just over a third believed a vaccination campaign would help prevent spread. Neither of these measures are appropriate for control of WNV since the horse is a dead-end host and subclinical infections are common (although vaccination protects the individual horse). The majority of remaining respondents answered, "I do not know" (Table 2).

There are inherent difficulties in using survey questions of this type, that is, using true or false responses for statements in this manner: responses may be biased towards agreement with the statements provided. It would have been preferable in this situation to use an open question; however, the decision was made to use true or false statements to act as a memory aid to participants, in case of partially recalled information. Particularly for these questions the authors assumed poor knowledge might be a problem, potentially leading to drop out of participants, or free text answers which could not be coded. By biasing this question towards producing apparently greater knowledge, it is possible to be more confident that lack of knowledge is unlikely to be overestimated, that is, even lower levels of knowledge are likely in the general population than reported. Ideally, further investigation of such complex questions would benefit from qualitative research methodology based on interviews.

A report by the Department for Environment, Food & Rural Affairs (DEFRA) on the risk of introduction of AHS into the country states that ’Awareness and familiarity of owners, keepers of horses and veterinarians with AHS clinical signs would facilitate early detection as a key limiting factor to potential wider dissemination of the disease in the UK’.^6^ While the majority of participants responded that AHS could cause death, only a small proportion gave specific clinical signs of AHS or WNV. This lack of specific knowledge is not surprising given that outbreaks of these diseases have never occurred in the UK but illustrates the need for dissemination of information to horse owners in the event of heightened disease risk. Online information on AHS is provided by The British Horse Society,^21^ the UK government^22^ and by individual veterinary practices, but based on the lack of knowledge in the horse owning community demonstrated by this study, investigation into horse owner engagement with these sources is warranted.

Surprisingly, a higher proportion of respondents in the UK (80.1 per cent) than in Kentucky, USA (66 per cent) said they would vaccinate against WNV even though the disease is endemic in Kentucky,^23^ and only around 40 per cent of UK leisure horses are vaccinated against influenza, which is present in the UK.^24^ However, respondents in the present study may have been biased towards horse owners with an interest in equine diseases and may, therefore, be more likely to vaccinate. The proportions of horse owners that stated they had their horses currently vaccinated is comparable with another UK horse owner survey in which 78.8 per cent and 87.6 per cent of respondents reported that their horses were vaccinated against influenza and tetanus, respectively.^25^ The main barriers to vaccination against WNV were potential side effects of a vaccine or the balance between side effects and efficacy. This would be important information to convey to veterinary surgeons, to promote discussion with horse owners and provide reassurance in the event of heightened disease risk to the UK.

Perceived efficacy of fly repellents by horse owners is likely to centre on general reduction in fly nuisance around horses. Therefore, the choice of repellent would probably be associated more with the aim of controlling flies such as biting midges and horse flies, rather than mosquitoes. Most study respondents stated that they currently seek information on bite-protection from their veterinary surgeon. Therefore, it is important that veterinary surgeons have evidence-based information in order to provide correct advice, particularly in a situation of heightened disease risk.^26 27^ Of the active ingredients used by respondents in this study, icaridin and DEET have both been shown to have some repellent effect for horses against mosquitoes.^28 29^ Evidence of the efficacy of active ingredients contained in specific products in this study are summarised in Table 3. However, it should be noted that many studies of repellent products show variable durations of effective protection against biting insects, few repellents have been tested in any livestock species and efficacy in human studies may not translate to protection for horses.^30^ Due to the small size of biting midges, direct testing of repellency for horses is challenging.^31^

There were some limitations to the study that are inherent in open, internet-based surveys. It was not possible to obtain a randomised representative sample of horse owners and therefore it was not possible to estimate a response rate. The inference that any questions left blank (unit non-response) were left intentionally blank may lead to bias; however, this was considered an acceptable trade-off as non-response due to lack of knowledge is common.^32^ It was considered that requiring responses in a survey investigating limited knowledge may lead to unacceptable loss of participants through forcing them to choose "I do not know", an answer they may not find comfortable. Sampling bias is also likely due to the requirement for internet access, and self-selection of horse owners with an interest in the topic of the questionnaire. Respondents may be biased towards the more media literate and, therefore, possibly, younger horse owner. Respondents were not asked for demographic information, such as their age or sex;however, a previous online survey of horse owners in Great Britain^33^ reported that 95.2 per cent of respondents were female and 51.6% were under 45 years old. The British Equestrian Trade Association’s National Equine Survey 2015^34^ reported that females represented 74 per cent of the riding population. The number of responses to this study is likely to have precluded meaningful analysis of demographic information. Respondents were not instructed to limit responses to one per household, so clustering of knowledge was possible. However, the majority of respondents supplied partial postcodes. Clustering within postcode districts was not apparent, and where only the town of residence was supplied, the maximum number of people giving the same town was two. This occurred in four locations.

As per the authors' knowledge, this is the first survey of UK horse owners that has been conducted to determine awareness of insects that transmit equine arboviruses, clinical signs of disease and control methods. While the majority of respondents were able to identify insect vectors such as mosquitoes and biting midges, most were unaware of diseases that they may transmit. In addition, most study respondents were unable to provide specific clinical signs of WNV and AHS, and few stated that these diseases may cause death of infected horses. A variety of methods were reported to be used to repel insects from biting horses, but in many cases, there is no published evidence of efficacy. The veterinary profession was stated as the key source of information about insect control and would be vital in the event of a disease outbreak in disseminating evidence about clinical signs of disease, methods of insect control and vaccination. Based on this study, should an equine arbovirus disease outbreak occur in northern Europe, it would be important for the UK veterinary profession to be able to quickly implement a horse owner education campaign and for veterinary surgeons to be able to provide accurate information about clinical signs and methods of disease prevention and control.
