# Supplementary material for: Survey of UK horse owners’ knowledge of equine arboviruses and disease vectors
Source: Vet Rec. 2018 May 15;183(5):159. doi: 10.1136/vr.104521 (PMC6089202; doi:10.1136/vr.104521)
Supplement: Supplementary file 1 [file vetrec-2017-104521supp001.pdf]

Horses, Insects and Infectious disease

Welcome

We are conducting a survey with owners/carers of horses in the UK about issues relating to insects biting horses and infectious disease risk. Even if you don’t feel that your horse has any problems with biting insects, we would like to hear from you. We are seeking information on attitudes to infectious disease, knowledge and awareness of particular diseases, and experiences of (or lack of) insect biting nuisance around your horse’s yard.

You can take part in this survey if you arecurrently caring for a horse, live in the UK and are over 16.

The project is funded by the Horse Trust and is carried out by a team of veterinary surgeons and epidemiologists at the University of Liverpool. The findings of this study aim to improve future information and advice about infectious disease for horse owners. The survey is completely anonymous. One of the questions asks for a partial postcode or approximate location of your horse’s yard – this is so that we can look for any regional variations across the UK and you can leave this answer blank if you wish.The information collected in the study will be maintained by Professor Debra Archer, University of Liverpool. (darcher@liverpool.ac.uk) If you have any further questions regarding the study please contact us: g.e.chapman@liv.ac.uk.

Insect 1 (Image attribution: CSIRO [CC BY 3.0 (http://creativecommons.org/licenses/by/3.0)], via Wikimedia Commons)

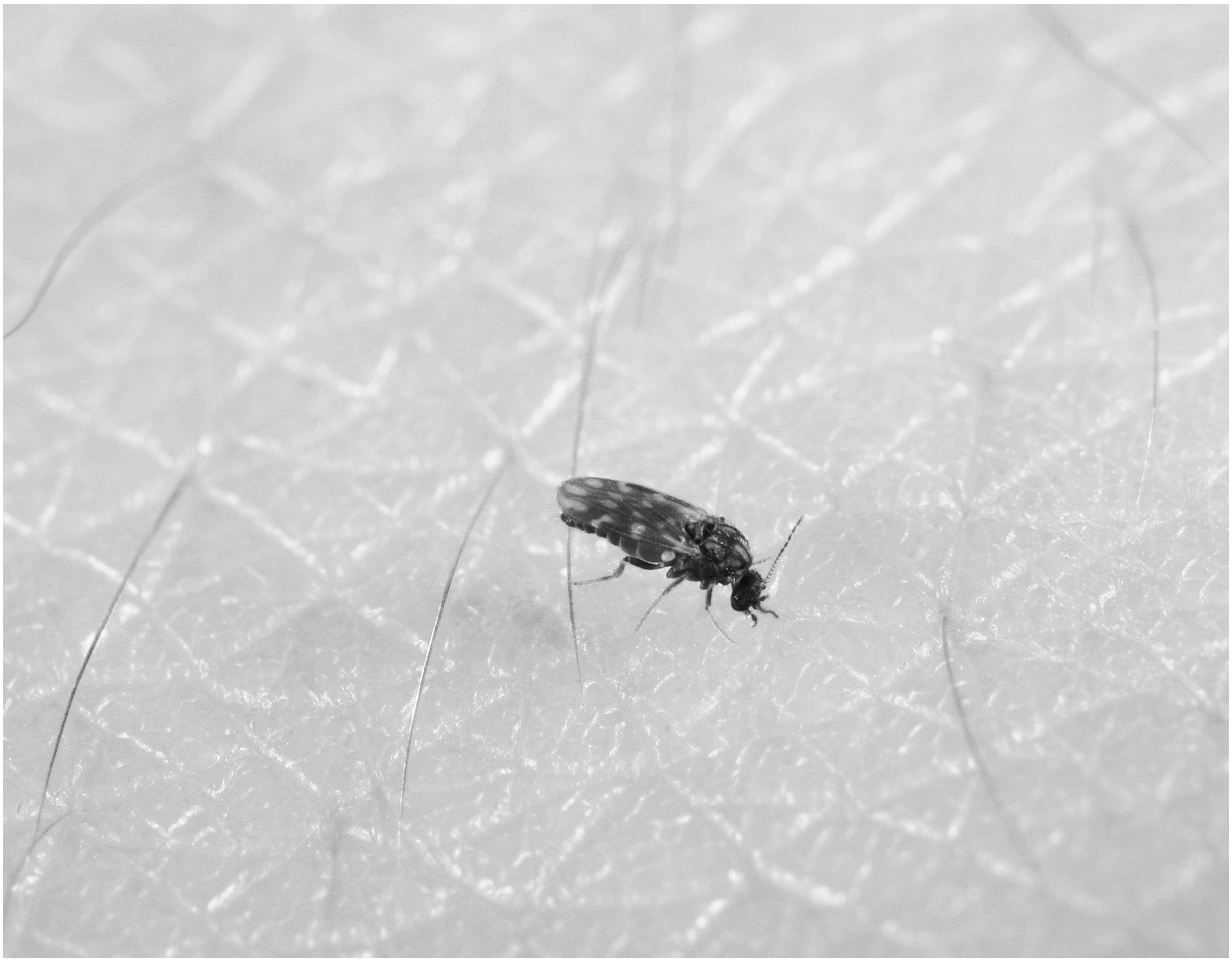

Insect 1 - Showing actual size

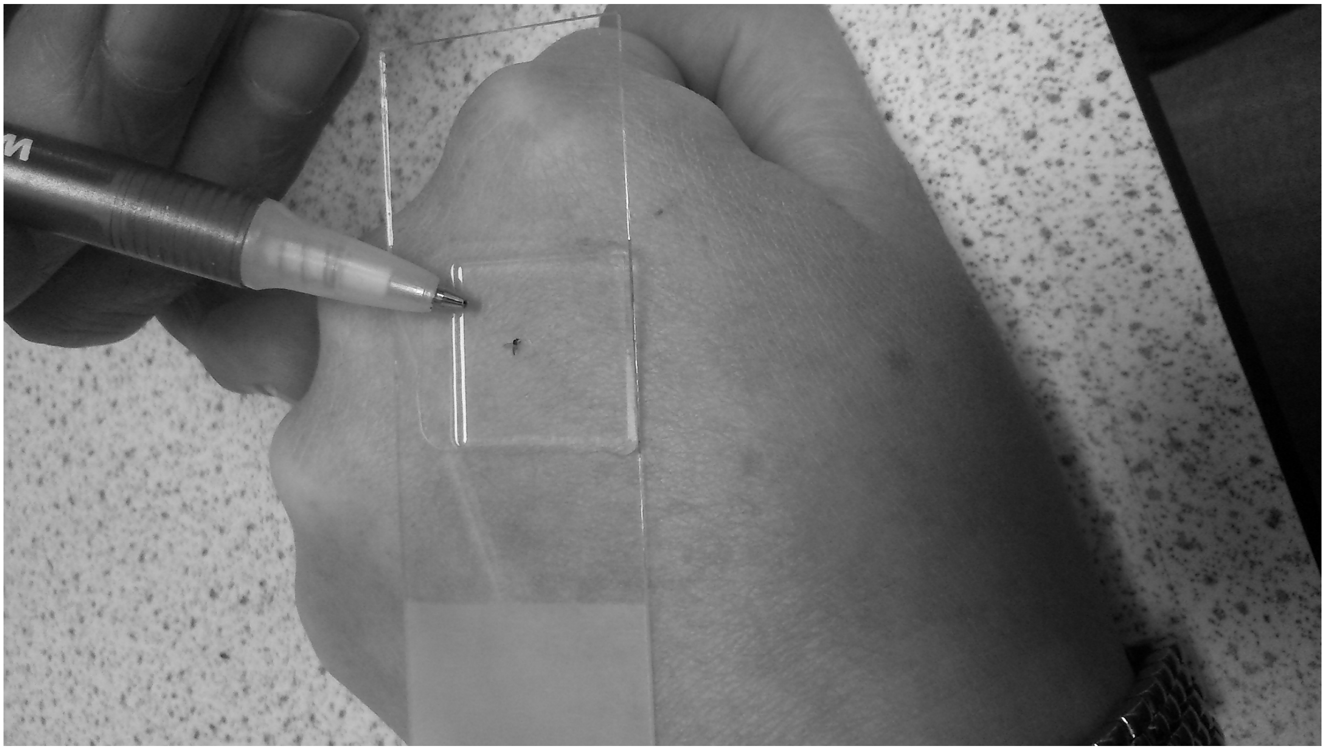

1. What would you call the insect above?

- ◯ Mosquito
- ◯ Midge
- ◯ Stable Fly
- ◯ Horse Fly
- ◯ Gnat
- ◯ I have never seen this before
- ◯ I do not know
- ◯ Other (please specify)

* 2. Do you believe that this type of insect causes disease (anywhere in the world)?

Insect 2 (Author: Abeer Jabbar. Available under public license https://www.flickr.com/photos/abeerjabbar/3296527723, http://creativecommons.org/licenses/by-sa/4.0)

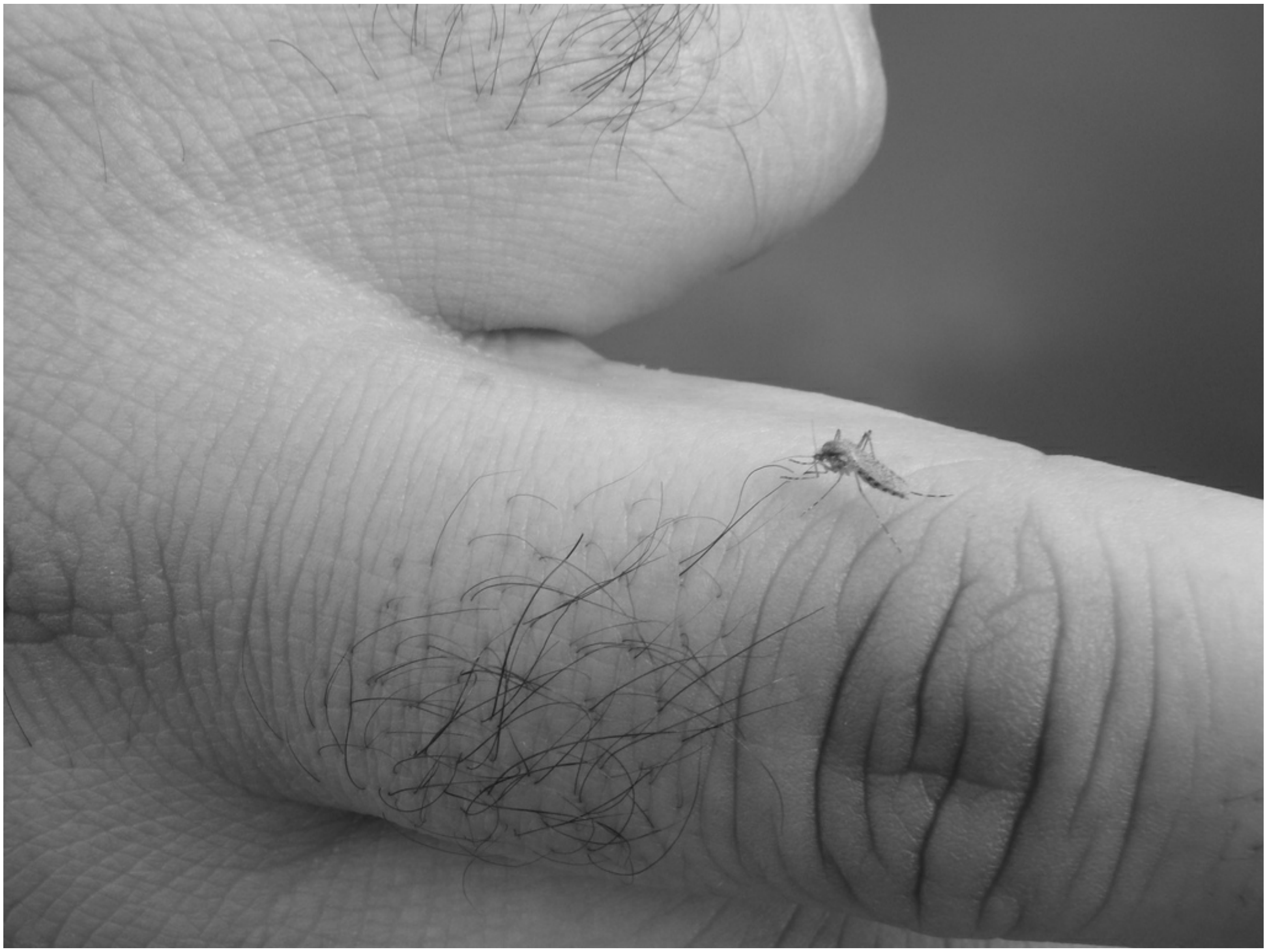

3. What would you call the insect above?

- ○ Mosquito
- ○ Midge
- ○ Stable Fly
- ○ Horse Fly
- ○ Gnat
- ○ I have never seen this before
- ○ I do not know
- ○ Other (please specify)

4. Do you believe that this type of insect causes disease (anywhere in the world)?

Insect 3

(Image attribution: By Fritz Geller-Grimm (Own work) [CC BY-SA 3.0 (http://creativecommons.org/licenses/by-sa/3.0), CC BY-SA 3.0 (http://creativecommons.org/licenses/by-sa/3.0), GFDL (http://www.gnu.org/copyleft/fdl.html) or CC BY-SA 3.0 (http://creativecommons.org/licenses/by-sa/3.0)], via Wikimedia Commons)

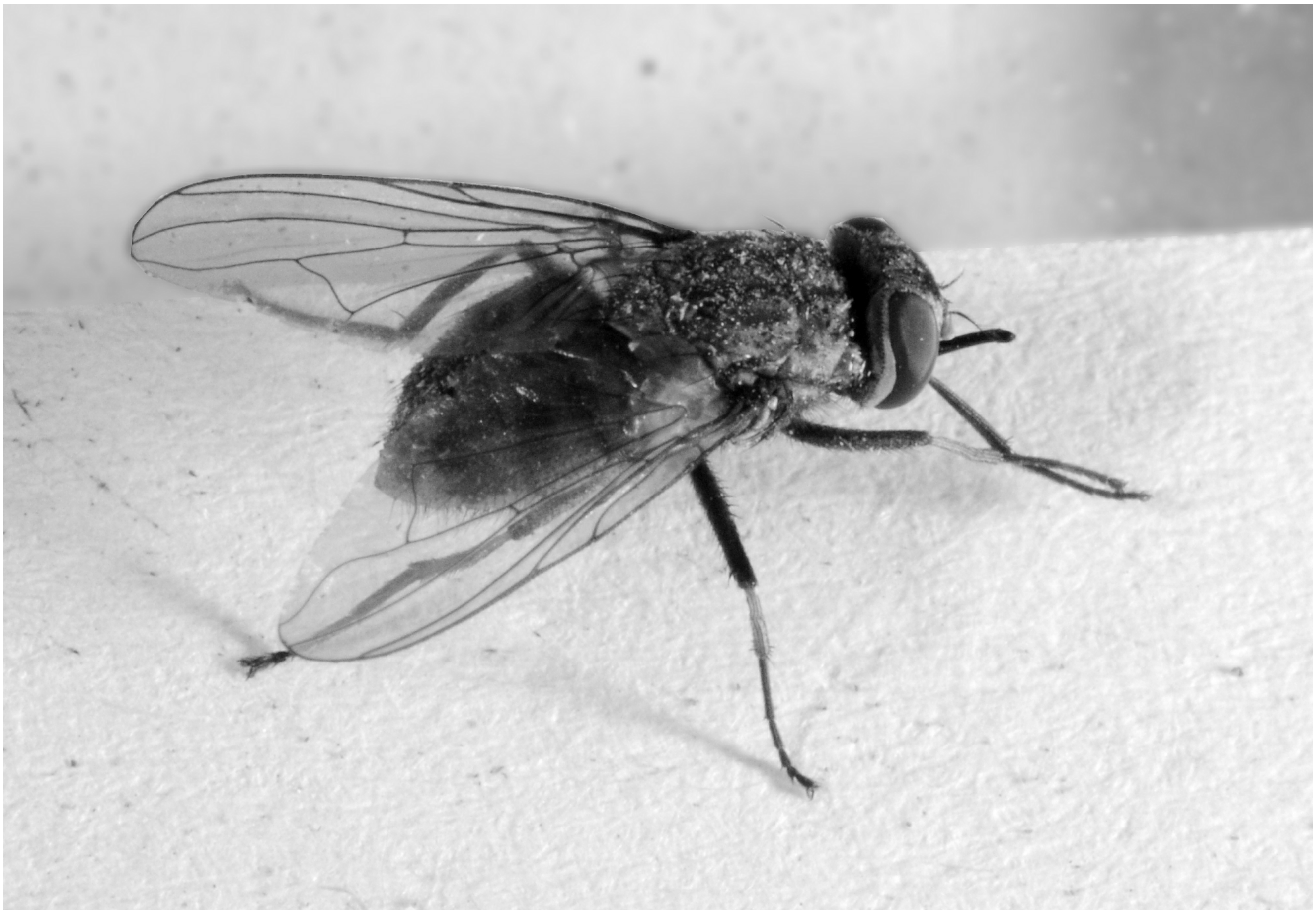

5. What would you call the insect above?

- ◯ Mosquito
- ◯ Midge
- ◯ Stable Fly
- ◯ Horse Fly
- ◯ Gnat
- ◯ I have never seen this before
- ◯ I do not know
- ◯ Other (please specify)

6. Do you believe that this type of insect causes disease (anywhere in the world)?

Insect 4

(Image attribution: By Sandy Rae from Scotland, UK [CC BY 2.0 (http://creativecommons.org/licenses/by/2.0)], via Wikimedia Commons)

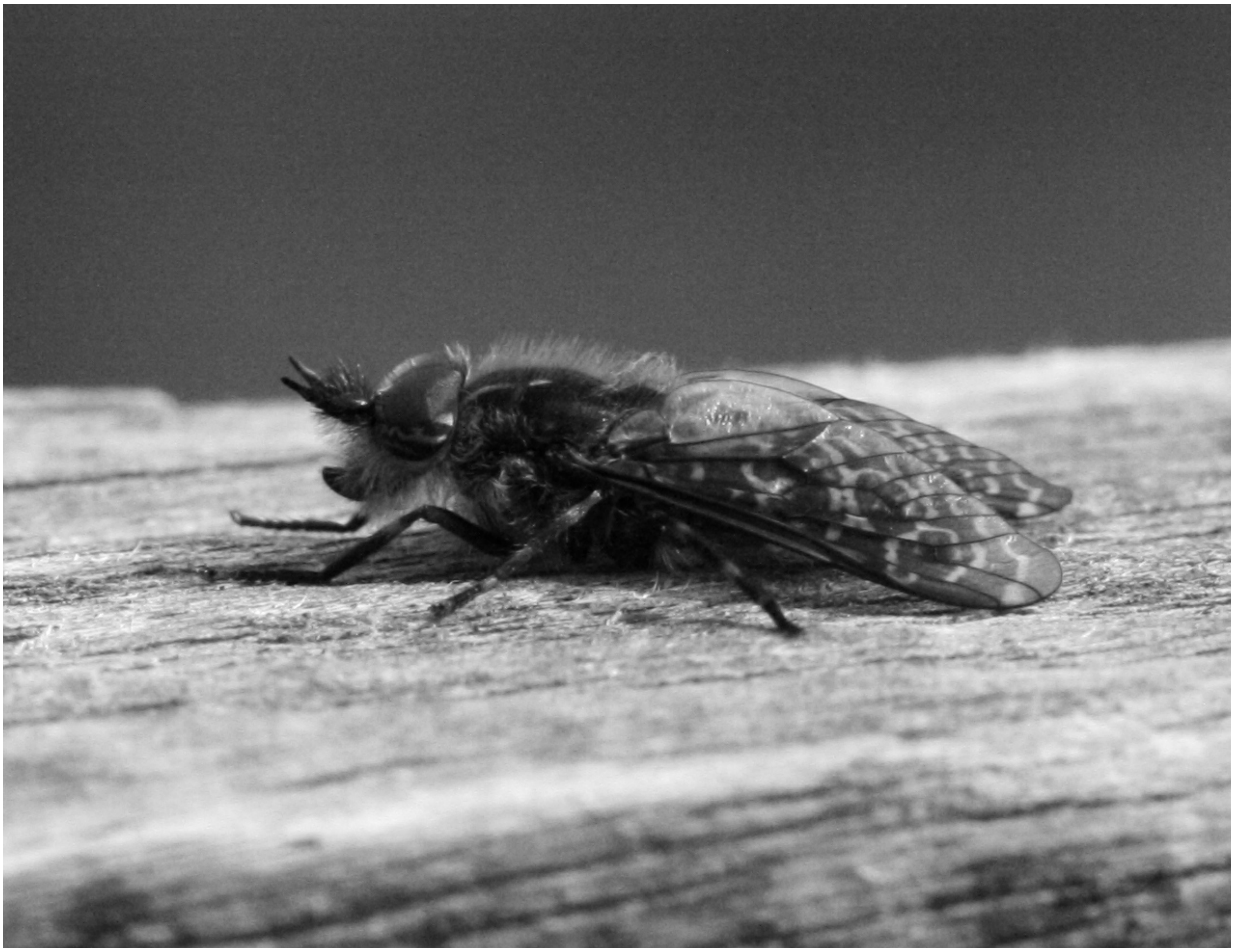

7. What would you call the insect above?

- ◯ Mosquito
- ◯ Midge
- ◯ Stable Fly
- ◯ Horse Fly
- ◯ Gnat
- ◯ I have never seen this before
- ◯ I do not know
- ◯ Other (please specify)

8. Do you believe that this type of insect causes disease (anywhere in the world)?

Horses, Insects and Infectious disease

9. Are you aware of biting insects on the premises where you keep your horse? (Check all that apply)

- ☐ Mosquitoes
- ☐ Midges
- ☐ Horse flies
- ☐ Stable flies
- ☐ Yes, but I'm not sure which
- ☐ Other biting insects (please specify)

10. What makes you aware of the presence of these insects on your yard (please check all that apply)

	MOSQUITOES	MIDGES
I am not aware of them	☐	☐
I see them on horses at pasture	☐	☐
I see them on horses in the stable	☐	☐
I see them biting horses	☐	☐
Horses are irritated by them	☐	☐
I get bitten	☐	☐
I see swarms of insects	☐	☐
Other	☐	☐

11. Do you feel that MOSQUITOES cause a problem on your yard, during the warmer months to any of the horses or people?

No	A minor problem	A moderate problem	A major problem
○	○	○	○

12. Do you feel that MIDGES cause a problem on your yard, during the warmer months to any of the horses or people?

No	A minor problem	A moderate problem	A major problem
○	○	○	○

13. Are there any diseases that you are aware of that MIDGES can pass to horses (worldwide)?

14. Are there any diseases that you are aware of that MOSQUITOES can pass to horses (worldwide)?

Horses, Insects and Infectious disease

15. Are you aware of West Nile Virus?

- ◯ Yes
- ◯ No

Horses, Insects and Infectious disease

16. Do you know if West Nile Virus can affect horses?

- ◯ Yes it can
- ◯ No it cannot
- ◯ I do not know

17. Have you heard of African Horse Sickness?

- ◯ Yes
- ◯ No

18. Are you aware of any other insect-borne diseases of horses (which occur anywhere in the world)?

- ◯ No
- ◯ Yes (please specify)

Horses, Insects and Infectious disease

19. Please check the box you believe is correct for each statement. Currently there are around 900,000 horses in the UK. If there were an outbreak of African Horse Sickness in the UK:

	True	False	I don't know
The disease could spread rapidly through the UK	◯	◯	◯
Many horses could become ill	◯	◯	◯
Horses could die from the disease	◯	◯	◯
Lots of horses (more than 1000) could die from the disease	◯	◯	◯
The government would ban movement of horses in affected areas	◯	◯	◯
A vaccination campaign would be necessary to prevent further spread	◯	◯	◯
Vaccination could be done immediately to protect horses	◯	◯	◯

20. Please check the box you believe is correct for each statement.
If there were an outbreak of West Nile virus in the UK:

	True	False	I don't know
The disease could spread rapidly through the UK	○	○	○
Many horses could become ill	○	○	○
Horses could die from the disease	○	○	○
Lots of horses (more than 1000) could die from the disease	○	○	○
The government would ban movement of horses in affected areas	○	○	○
A vaccination campaign would be necessary to prevent further spread	○	○	○
Vaccination could be done immediately to protect horses	○	○	○

21. Please describe what signs you believe a horse might show if it was infected with West Nile Virus. If you are not aware of any at all, leave blank.

22. Please describe what signs you believe a horse might show if it was infected with African Horse Sickness. If you are not aware of any at all, leave blank.

Horses, Insects and Infectious disease

23. There is a vaccine for West Nile virus. Assuming it was priced around £30-£35 per vaccine, similar to a combined flu and tetanus vaccine - (2 vaccinations in the first year then a booster each year) and you would pay your usual veterinary call out fee, would you have your horse vaccinated if an outbreak of disease occurred in the UK?

- ◯ Yes
- ◯ No
- ◯ Maybe

24. What might stop you from having your horse vaccinated?

25. Do you routinely have your horse(s) vaccinated for:

- ☐ Influenza?
- ☐ Tetanus?
- ☐ Other (please specify)

Horses, Insects and Infectious disease

26. Please indicate if any of these applies to your horse, or to ALL of your horses, if you have more than one:

- [ ] My horse is / horses are not ridden / driven
- [ ] My horse is / horses are at pasture permanently and rarely use a stable (please check this even if they use a field shelter)
- [ ] My horse is / horses are never or rarely kept in the stable in the summer months (e.g. just to tack up, wait for farrier etc)

These are not relevant as my horse's management do not fit these criteria. (Please describe your horse's management).

27. Do you use any of these methods to reduce insect bites to your horse? (Check all that apply)

	In the stable	At pasture	Only when ridden / driven
Apply repellent when the horse is:	☐	☐	☐
Use fly rugs / exercise sheet when the horse is:	☐	☐	☐
Use fly masks or fringes when the horse is:	☐	☐	☐

28. Do you use any of these methods to reduce insect bites to your horse? (Check all that apply)

- [ ] Barriers such as fly screens to prevent insects entering the stable
- [ ] Wash in insecticides / repellents such as Deosect, Coopers Fly Repellent Plus, or similar
- [ ] None of the these
- [ ] Spray on Fly repellent - Please specify which product(s) you use.

29. If you use any other methods to reduce insect bites, or would like to comment on how you combine methods for increased protection, please describe here:

30. Are there any bite reduction methods that you feel are useful against one insect but not another. If so please describe:

31. Who would you currently ask for advice on insect control in relation to your horse? (Check all that apply)

- [ ] Veterinary surgeon
- [ ] Yard Owner
- [ ] Tack shop staff
- [ ] Others on your yard
- [ ] Internet sources
- [ ] Other (please specify)

32. If you answered that you use the internet in the previous question, please describe which sources you would use to find out about protecting horses insect bites

Horses, Insects and Infectious disease

33. If there were an outbreak of insect-borne disease in the UK, would you seek information about disease control, or protection from insects from a different source?

- No
- Yes - Please give your thoughts on this

34. Do you have any other comments about insect-borne disease?

35. If you are happy to do so, please supply the first four digits of the postcode, or nearest town and the county of your yard.

Horses, Insects and Infectious disease

Thank you for completing this survey

Thank you for completing our survey. If you would like further information about mosquito-borne viruses or the project it is available here.
Please remember that no horse has ever caught any of these diseases in the UK, and none of the diseases mentioned in this survey or considered in this project is currently present. Also, there are vaccinations available for the majority of these diseases in countries affected. If you have any further questions or concerns regarding these diseases you are welcome to contact us: g.e.chapman@liv.ac.uk.
